# Real-World Assessment of Clinical Outcomes in Patients with Lower-Risk Myelofibrosis Receiving Treatment with Ruxolitinib

**DOI:** 10.1155/2015/848473

**Published:** 2015-11-09

**Authors:** Keith L. Davis, Isabelle Côté, James A. Kaye, Estella Mendelson, Haitao Gao, Julian Perez Ronco

**Affiliations:** ^1^RTI Health Solutions, Research Triangle Park, NC 27709, USA; ^2^Novartis Pharmaceuticals Corporation, East Hanover, NJ 07936, USA; ^3^RTI Health Solutions, Waltham, MA 02451, USA; ^4^Novartis Pharma AG, 4056 Basel, Switzerland

## Abstract

Few trial-based assessments of ruxolitinib in patients with lower-risk myelofibrosis (MF) have been conducted, and no studies have made such assessments in a real-world population. We assessed changes in spleen size and constitutional symptoms during ruxolitinib treatment using a retrospective, observational review of anonymized US medical record data of patients diagnosed with IPSS low-risk (*n* = 25) or intermediate-1-risk (*n* = 83) MF. The majority of patients were male (low risk, 60%; intermediate-1 risk, 69%). Most patients (92% and 77%) were still receiving ruxolitinib at the medical record abstraction date (median observation/exposure time, 8 months). The proportion of patients with moderate or severe palpable splenomegaly (≥10 cm) decreased from diagnosis (56%) to best response (12%). Fatigue was reported in 47% of patients and was the most common constitutional symptom. For most symptoms in both risk groups, shifts in the distribution of severity from more to less severe from diagnosis to best response were observed. Both patients with low-risk and intermediate-1-risk MF experienced a substantial decrease in spleen size with ruxolitinib treatment in real-world settings. For most symptoms examined, there were distinct improvements in the distribution of severity during ruxolitinib treatment. These findings suggest that patients with lower-risk MF may benefit clinically from ruxolitinib treatment.

## 1. Introduction

Myelofibrosis (MF) is a myeloproliferative neoplasm (MPN) characterized by cytopenias, splenomegaly, constitutional symptoms (e.g., fatigue, early satiety, weight loss, night sweats, fever, bone pain, and pruritus), and progressive bone marrow fibrosis [1]. It is a chronic disease that reduces life expectancy and quality of life [2]. MF is rare, with an incidence in the United States of 0.2 per 100,000 persons [3]. Survival in patients with MF is highly variable [4, 5], depending on the presence of specific prognostic factors such as those incorporated in the International Prognostic Scoring System (IPSS; Table 1). Median survival has been estimated at 11 years for patients with IPSS low-risk MF, 8 years for intermediate-1-risk MF, 4 years for intermediate-2-risk MF, and 2 years for high-risk MF [4].

Until recently, medical and surgical options for patients with MF have been limited [6]. Most pharmacotherapies were palliative [7], and their effect on spleen size and symptoms was minimal and generally of short duration [8, 9]. Splenectomy may be considered for patients with substantial spleen enlargement and/or refractory splenic symptoms that have not responded to pharmacotherapy [10]; however, mortality and morbidity rates associated with splenectomy (9% and 31%, resp.) are significant and limit its therapeutic use [11]. For patients with symptomatic splenomegaly who are not candidates for surgery, splenic irradiation may be offered; however, its benefit (mainly palliative) is often short lived and patients may experience significant toxicities [10]. Although allogeneic stem cell transplant is the only treatment with curative potential [12], it carries significant risks of morbidity and mortality [13], particularly in older patients; therefore, few patients with MF are suitable candidates for this approach [7].

The recent identification of mutations associated with the Janus kinase (JAK)/signal transducer and activator of transcription (STAT) pathway and a new appreciation of the role of cytokines signaled through JAK1 and JAK2 in the pathogenesis of MPNs [14–16] has resulted in new treatment strategies for these diseases. Based on this new knowledge, a selective, orally available JAK inhibitor (ruxolitinib) has been developed for the treatment of MF. Randomized clinical trials of ruxolitinib demonstrated reductions in splenomegaly and MF-related symptoms that led to US regulatory approval of the drug [8, 17] for patients with IPSS intermediate- and high-risk MF [4] and European market authorization for patients with MF-related splenomegaly or symptoms.

To date, few trial-based assessments of ruxolitinib in patients with lower-risk MF have been conducted, and no studies have made such assessments in a real-world setting. In this study, we sought to understand whether symptomatic patients with lower-risk MF would also benefit from ruxolitinib treatment, as was seen in patients with intermediate-2- and high-risk disease studied in the registration trials, by retrospectively assessing changes in spleen size and disease-related symptoms in routine clinical practice.

## 2. Methods

This was a retrospective, observational review of anonymized medical record data collected in January 2014 by 49 hematologists and oncologists in the United States. Participating physicians were recruited from an existing research panel maintained by All Global, Ltd. Data were collected with secure, online case report forms (CRFs) administered to the selected physicians who had treated ≥2 patients with MF with ruxolitinib since November 2011 (US launch date for the drug). Patient inclusion criteria were as follows: (1) being diagnosed with lower-risk MF (IPSS score of 0 [low risk] or 1 [intermediate-1 risk]); (2) being first treated with ruxolitinib ≥3 months before the medical record abstraction date; (3) being ≥18 years of age at ruxolitinib initiation; (4) having a medical history from MF diagnosis until the medical record abstraction date; and (5) never being enrolled in an MF-related interventional trial. Minimum quotas of 25 and 50 were set for patients with low- and intermediate-1-risk disease, respectively, with a predetermined maximum of 110 patients in the combined total. To increase generalizability of the sample, each physician was limited to 3 patient entries (although most physicians entered only 2). Furthermore, when >3 patient records were available for a physician, patients with birth months nearest to a birth month randomly generated by the electronic CRF were selected.

Spleen size and constitutional symptoms were the key measures, retrospectively extracted at MF diagnosis, at ruxolitinib initiation, and at best response while receiving ruxolitinib treatment. Symptoms of interest included those described in the validated Myeloproliferative Neoplasm Symptom Assessment Form (MPN-SAF) [18], which were categorized as mild, moderate, or severe based on medical notes recorded at each time point. Symptom data were collected only to the extent that they were documented in the patient medical records; patients were not contacted by their physicians or other study personnel to obtain this information. For this analysis, we present findings on the 7 most commonly observed MPN-SAF symptoms in our sample (full tabular results on all 17 MPN-SAF symptoms are available upon request). Spleen size was captured using predefined categories of no splenomegaly present (spleen not palpable), very mild or mild splenomegaly (<10 cm palpated), moderate splenomegaly (10–20 cm palpated), or severe splenomegaly (>20 cm palpated).

Although this study was not designed as a safety evaluation of ruxolitinib, we additionally report the frequency of thrombocytopenia and anemia, which are the 2 most common adverse events associated with ruxolitinib (as expected based on the drug's mechanism of action), as well as the proportion of patients who required a change in ruxolitinib treatment (i.e., dose reduction, temporary therapy interruption, or therapy discontinuation) as a result of an adverse reaction. Following previous safety reporting from the ruxolitinib COMFORT-I trial [17], we specifically report the proportion of patients experiencing grade ≥3 thrombocytopenia (platelet count <50 × 10^9^/L) or grade ≥3 anemia (hemoglobin <8 g/dL) as measured at any point after ruxolitinib initiation through last ruxolitinib dose.

All statistical analyses were carried out using SAS (version 9.3; SAS Institute Inc., Cary, NC, USA) statistical software. Because this study was exploratory in nature with no proposed hypotheses to test, only descriptive analyses were implemented. These analyses entailed the tabular display of means, SDs, medians, and value ranges for continuous variables and the frequency distribution of categorical variables. Conduct of this study was approved by an authorized institutional review board (Research Triangle Institute Committee for the Protection of Human Subjects, Federal Wide Assurance #3331) and carried out in accordance with the 1996 Helsinki Declaration regarding the ethical conduct of human subject research. Because anonymized retrospective patient data were collected, an informed consent waiver was granted.

## 3. Results

A total of 49 physicians were recruited for the data abstraction, of whom 82% specialized in hematology/oncology. Mean (SD) duration of practice experience was 12.7 (5.9) years, and the majority (73%) of the physicians practiced in community-based group clinics. A total of 108 patients were included (25 with low-risk and 83 with intermediate-1-risk disease) in this study (Table 2 shows summarized data on key characteristics of these patients). All 25 patients with low-risk and nearly 80% of those with intermediate-1-risk MF were aged ≤65 years. The majority of patients in both risk groups were male (60% for low risk and 69% for intermediate-1 risk). Most patients in both risk groups (80% for low risk and 82% for intermediate-1 risk) had primary MF at initial diagnosis. A substantially higher proportion of the patients with intermediate-1-risk disease were positive for the* JAK2* V617F mutation (72%) compared with patients with low-risk disease (56%). The prevalence of comorbidities in the selected patients appeared to be consistent with that in the general population, with diabetes and hypertension the most common conditions recorded at ruxolitinib initiation. Finally, most patients in both risk groups (92% for low risk and 77% for intermediate-1 risk) were still receiving ruxolitinib treatment at the time of data abstraction or last available follow-up.

### 3.1. Spleen Size

**Figure 1:**
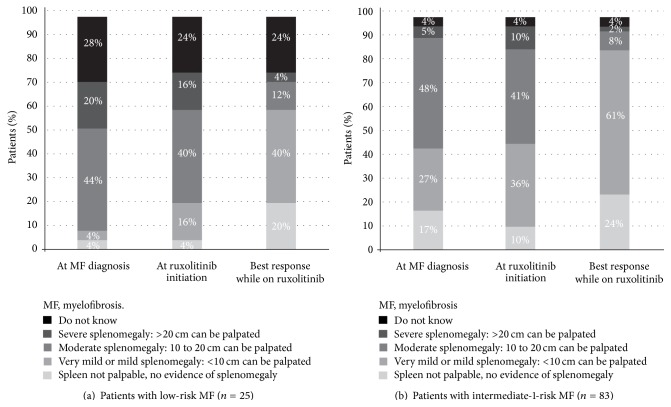
Spleen size distribution.

Based on patients' best treatment response, Figure 1 shows that patients with low-risk disease experienced a substantial improvement in spleen size during ruxolitinib treatment compared with the recorded spleen size at MF diagnosis and at ruxolitinib initiation. Specifically, the combined proportion of patients with low-risk MF with moderate or severe splenomegaly decreased from 64% at MF diagnosis to 16% at best response during ruxolitinib treatment. Likewise, the combined proportion of patients with low-risk disease with either no evidence of splenomegaly or mild splenomegaly increased from 8% at MF diagnosis to 60% at best response during ruxolitinib treatment. Overall, 78% of patients with low-risk disease had a decrease in spleen size from MF diagnosis to best response during ruxolitinib treatment, and 68% of patients had a decrease from ruxolitinib initiation to best response. Similar findings were obtained for patients with intermediate-1-risk MF: the combined proportion of patients with intermediate-1-risk disease with moderate or severe splenomegaly decreased from 53% at MF diagnosis to 10% at best response during ruxolitinib treatment, whereas the combined proportion of patients with either no evidence of splenomegaly or only mild splenomegaly increased from 44% at MF diagnosis to 85% at best response during ruxolitinib treatment. Similar to the low-risk population, 55% of patients with intermediate-1-risk disease had a decrease in spleen size from MF diagnosis to best response during ruxolitinib treatment, and 55% of those patients had a decrease from ruxolitinib initiation to best response.

### 3.2. Symptoms

**Figure 2:**
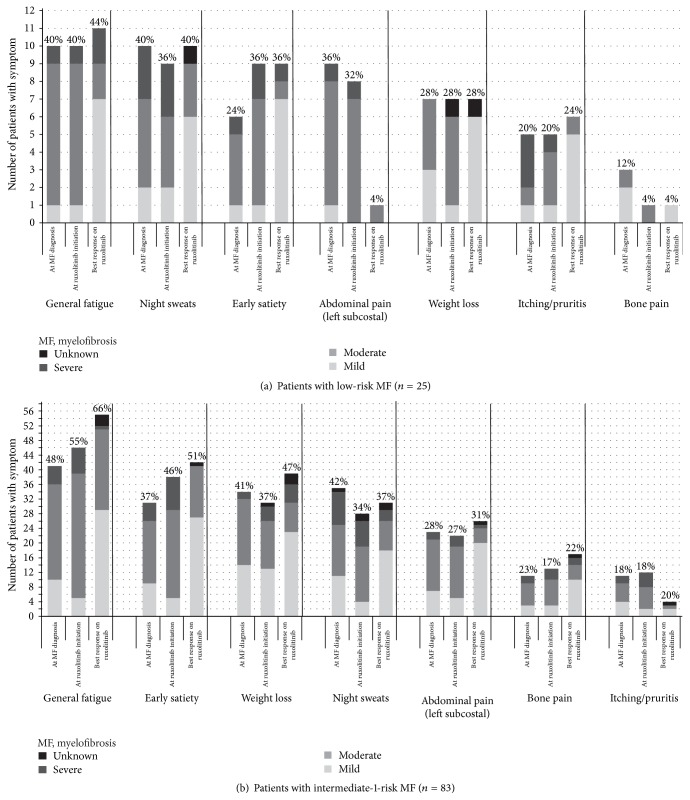
Symptom frequency and severity distribution.

In general, for both risk groups, a distinct shift was observed in the distribution of symptom severity toward a more favorable profile (i.e., less severe) from MF diagnosis to the time of best response during ruxolitinib treatment (Figure 2). Among patients with low-risk MF with fatigue, for example, the proportion with moderate or severe fatigue decreased from 90% at MF diagnosis to 37% at best ruxolitinib response; in patients with intermediate-1-risk disease, the decrease was from 76% at MF diagnosis to 42% at best response. However, the number of patients still experiencing each symptom, even though experiencing it in a less severe form for the majority of the symptoms, did not decrease for all symptoms examined. For patients with low-risk disease, general fatigue, night sweats, and early satiety were the 3 most common symptoms, experienced by one-third to nearly one-half of patients, depending on the observation point and symptom examined. For patients with intermediate-1-risk disease, general fatigue, night sweats, and weight loss were the 3 most common symptoms, reported in approximately one-half to two-thirds of patients.

### 3.3. Adverse Events

Grade ≥ 3 thrombocytopenia was observed in 7% of all patients at some point during ruxolitinib treatment (12% of low-risk patients and 6% of intermediate-1-risk patients); grade ≥ 3 anemia was observed in 22% of patients (20% of low-risk patients and 23% of intermediate-1-risk patients) (Table 3). A reduction in ruxolitinib dose due to an adverse reaction was documented in 18% of all patients (12% of low-risk patients and 19% of intermediate-1-risk patients). Temporary therapy interruption and/or discontinuation were rare, only 1 reported case of each event (both events were observed in low-risk patients).

## 4. Conclusions

In light of robust trial data showing that ruxolitinib improved both splenomegaly-related and non-splenomegaly-related constitutional symptoms in patients with intermediate-2-risk and high-risk MF [8, 17], the present study sought to explore whether patients with MF in lower-risk prognostic categories may also benefit from treatment with ruxolitinib in a routine clinical setting. Our findings indicated that patients with lower-risk MF may indeed benefit from ruxolitinib, particularly with regard to splenomegaly reduction and improvement in both splenomegaly-related and constitutional symptoms. Based on patients' best treatment response, both patients with low-risk MF and those with intermediate-1-risk MF experienced a substantial improvement in spleen size during ruxolitinib treatment compared with the recorded spleen size at MF diagnosis and at ruxolitinib initiation. It is important to note that the reductions in spleen size reported here may be a conservative estimate of the maximum spleen size reduction each patient experienced during ruxolitinib treatment because the majority of patients were still on ruxolitinib at last follow-up; with longer follow-up, it is possible that an even more favorable response would have been observed, but additional research in patients with longer ruxolitinib exposure is needed to evaluate this. Furthermore, for most commonly occurring symptoms, we observed a distinct shift in the severity distribution toward a more favorable profile (i.e., less severe) from MF diagnosis to the time of best response during the observed duration of treatment.

To our knowledge, only 1 previous study [19] sought to assess in a clinical trial setting the possible therapeutic benefits of ruxolitinib in patients with lower-risk MF. These data from the ROBUST trial (ClinicalTrials.gov NCT01558739) in the United Kingdom showed that half of the patients with intermediate-1-risk MF treated with ruxolitinib achieved a reduction in spleen size of ≥50% at week 48 (versus baseline) after initiation of ruxolitinib. Mead et al. [19] also reported improvements in disease-related symptoms, as assessed using the Myelofibrosis Symptom Assessment Form (MF-SAF), for more than half (57%) of patients with intermediate-1-risk disease treated with ruxolitinib. Taken together, these findings are consistent with those reported for the routine clinical setting from which our study data were collected.

Although the study by Mead et al. [19] represents the only trial-based assessment to date of the clinical benefits of ruxolitinib in patients with lower-risk MF, our study remains, to our knowledge, the only such reporting from a clinical practice setting that stratified lower-risk patients based on the IPSS classification system. One previous study [20] reported on symptom improvement in the first month of ruxolitinib therapy for a small cohort of patients (*n* = 6) without splenomegaly at MF diagnosis. To the extent that absence of splenomegaly is a proxy for lower-risk MF, a comparison of findings on symptom improvement between that study and ours may be useful. Benjamini et al. [20] found a significant improvement in fatigue in all patients. Drenching night sweats (2 patients), itching (2 patients), and bone pain and skin rash thought to be paraneoplastic (1 patient) were also observed to resolve. As in that study, we observed improvements in fatigue, night sweats, itching, and bone pain for patients with intermediate-1-risk disease; we did not directly evaluate skin rash. Another study reporting the first postmarketing clinical experience with ruxolitinib (*N* = 28) found substantial improvements in constitutional symptoms and in spleen size [21]. However, although more than half of the patients in this study were intermediate-1 risk, the study did not present results by risk category, and no patients with low-risk MF were included in the analysis.

In our study sample, we also found that more than half (53%) of patients with intermediate-1-risk MF had moderate to severe splenomegaly (palpable spleen > 10 cm) at MF diagnosis. Two previous medical record reviews [22, 23] also indicated the possibility of a considerable rate of moderate to severe splenomegaly, even in patients with lower-risk MF. These studies, however, examined pooled cohorts of patients with both lower-risk (low and intermediate-1 risk) and higher-risk (intermediate-2 and high risk) patients and did not stratify splenomegaly frequency by risk category at diagnosis. Nonetheless, in the pooled cohorts (*n* = 74 in Benites et al. [22] and *n* = 1000 in Tefferi et al. [23]), at least moderate splenomegaly was found in 42% [22] and 21% [23] of patients. Because these samples comprised both patients with higher- and lower-risk disease, our finding of a 53% moderate to severe splenomegaly rate in patients with intermediate-1-risk MF (the rate was 66% in our small sample of patients with low-risk disease) might seem high. However, as previously noted, our study's data collection effort targeted hematologists and oncologists with experience prescribing ruxolitinib, and therefore we may inadvertently have studied a patient population that may have been inherently more complex (i.e., with higher rates of splenomegaly and constitutional symptoms) or more thoroughly evaluated than the patients seen in more general hematology/oncology practice settings. Despite this caveat, our findings, combined with those of the noted studies by Benites et al. [22] and Tefferi et al. [23], indicated that some degree of symptomatic splenomegaly was present in many patients with MF of all risk categories, which further supports the conclusion that ruxolitinib may address an unmet medical need in patients with lower-risk disease, as well as those for whom ruxolitinib treatment is currently indicated.

Our findings on the 2 most common adverse events associated with ruxolitinib (thrombocytopenia and anemia) were consistent with safety data reported in the ruxolitinib COMFORT-I trial [17], in which rates of grade ≥3 thrombocytopenia peaked at 6% at week 8 of treatment and those of grade ≥3 anemia peaked at 26% at week 8. In our study, we found that approximately 7% of patients had ≥1 occurrence of grade ≥3 thrombocytopenia at any point during ruxolitinib treatment, while 22% had ≥1 occurrence of anemia during treatment. Although assessments of these adverse events in the COMFORT-I study were protocol driven in that they were made at frequent predefined intervals, findings from our study (in which assessments were likely made less frequently and not at predefined intervals) appear to be consistent with the COMFORT-I results. In line with the anticipated occurrences of thrombocytopenia and anemia, we observed that nearly 18% of patients had a reduction in ruxolitinib dose due to an adverse reaction; complete discontinuation of ruxolitinib treatment due to an adverse reaction occurred in only 1 patient. Moreover, 12% of patients in our study received a red blood cell transfusion during ruxolitinib treatment to treat anemia (tabular data available upon request). These findings indicate, as described in a recent review article by Mesa and Cortes [24] in the context of a trial-based population, that hematologic events in real-world settings in patients treated with ruxolitinib can be successfully managed with dose modifications and red blood cell transfusions (in the case of anemia) and, importantly, are seldom reason for permanent treatment discontinuation.

This study is subject to several limitations. As in many retrospective medical record abstraction studies, patients selected for inclusion represent a convenience sample. Our study findings therefore may not be generalizable to the overall low- or intermediate-1-risk MF populations in the United States, and although participating physicians were recruited from all geographic regions, it was not possible to construct sampling weights that allowed for generalization to the national population. Only physicians who agreed to participate in the study contributed data; these physicians therefore may not be representative of all physicians treating low-risk or intermediate-1 MF in the United States. Finally, although no time limit was imposed on physicians for the completion of individual CRFs, the CRF was designed to limit physicians' time burden to help ensure full and accurate responses. Therefore, the scope of information that could be collected in this study was limited, and it is possible that additional information could have contributed further context to the study findings.

Despite the noted limitations, findings from this study indicated that patients with lower-risk MF in routine clinical practice may benefit from ruxolitinib treatment, specifically for spleen size reduction and improved splenomegaly-related and constitutional symptoms. Furthermore, ruxolitinib has been shown to prolong overall survival in patients with intermediate-2 or high-risk MF and to reduce the risk of death among high-risk patients receiving ruxolitinib to that of intermediate-2-risk patients receiving placebo or best available therapy [25]. These results suggest a potential to alter the clinical course of patients with MF and strongly support further evaluation of the effect of ruxolitinib in patients with intermediate-1 or low-risk MF. Data presented in this study, in conjunction with additional clinical trials, may also be useful in economic (e.g., cost-effectiveness) assessments of ruxolitinib use in patients with lower-risk MF.

## Figures and Tables

**Table 1:** International Prognostic Scoring System.

Variable (1 point each)	Risk group
Age > 65 years	Low risk: 0 points
Constitutional symptoms	Intermediate-1 risk: 1 point
Hemoglobin < 10 g/dL	Intermediate-2 risk: 2 points
Leukocyte count > 25 × 10^9^/L	High risk: ≥ 3 points
Circulating blasts ≥ 1%	

**Table 2:** Patient characteristics.

	All patients		IPSS category			
			Low risk		Intermediate-1 risk	
	*n*	%	*n*	%	*n*	%
Total patients	108	100.00	25	100.00	83	100.00
Age						
≤65 years	91	84.26	25	100.00	66	79.52
>65 years	17	15.74	0	0.00	17	20.48
Sex						
Male	72	66.67	15	60.00	57	68.67
Female	36	33.33	10	40.00	26	31.33
Race or ethnicity						
White	79	73.15	21	84.00	58	69.88
Black	16	14.81	2	8.00	14	16.87
Hispanic	10	9.26	0	0.00	10	12.05
Other	2	1.85	1	4.00	1	1.20
Do not know	1	0.93	1	4.00	0	0.00
Primary insurance type at ruxolitinib initiation						
Commercial	43	39.81	10	40.00	33	39.76
Medicare	47	43.52	9	36.00	38	45.78
Medicaid	9	8.33	2	8.00	7	8.43
Uninsured	0	0.00	0	0.00	0	0.00
Other	2	1.85	0	0.00	2	2.41
Do not know	7	6.48	4	16.00	3	3.61
MF type at diagnosis						
Primary MF	88	81.48	20	80.00	68	81.93
Postpolycythemia vera MF	10	9.26	3	12.00	7	8.43
Postessential thrombocythemia MF	9	8.33	2	8.00	7	8.43
Do not know	1	0.93	0	0.00	1	1.20
*JAK2* V617F mutation test result						
Positive	74	68.52	14	56.00	60	72.29
Negative	21	19.44	7	28.00	14	16.87
Test not done	3	2.78	1	4.00	2	2.41
Do not know	10	9.26	3	12.00	7	8.43
Charlson comorbidities at ruxolitinib initiation						
Hypertension	35	32.41	6	24.00	29	34.94
Diabetes (overall)	17	15.74	2	8.00	15	18.07
Diabetes (without end organ damage)	15	13.89	1	4.00	14	16.87
Chronic pulmonary disease	9	8.33	1	4.00	8	9.64
Liver disease (overall)	7	6.48	1	4.00	6	7.23
Depression	7	6.48	0	0.00	7	8.43
Mild liver disease	6	5.56	1	4.00	5	6.02
Cerebrovascular disease	5	4.63	1	4.00	4	4.82
Connective tissue disease	4	3.7	1	4.00	3	3.61
Dementia	3	2.78	0	0.00	3	3.61
Malignant solid tumor	3	2.78	1	4.00	2	2.41
HIV/AIDS	2	1.85	0	0.00	2	2.41
Hemiplegia	2	1.85	0	0.00	2	2.41
Myocardial infarction	2	1.85	0	0.00	2	2.41
Diabetes (with end organ damage)	2	1.85	1	4.00	1	1.20
Malignant lymphoma	1	0.93	0	0.00	1	1.20
Moderate or severe liver disease	1	0.93	0	0.00	1	1.20
Peripheral vascular disease	1	0.93	0	0.00	1	1.20
Ulcer disease	1	0.93	1	4.00	0	0.00
Congestive heart failure	0	0	0	0.00	0	0.00
None of these	36	33.33	11	44.0	25	30.12
Other	0	0.00	0	0.0	0	0.00
Do not know	8	7.41	4	16.0	4	4.82
Ruxolitinib doses utilized						
Starting median daily dose, mg (min, max)	30 (2, 56)		30 (4, 56)		30 (2, 50)	
Dose range observed over entire treatment duration, *n* (min, max)	2, 60		4, 60		2, 50	
Still on ruxolitinib at last available follow-up?						
Yes	87	80.60	23	92.00	64	77.10
No	15	13.90	2	8.00	13	15.70
Do not know	6	5.60	0	0.00	6	7.20

IPSS, International Prognostic Scoring System; JAK2, Janus kinase 2; MF, myelofibrosis.

**Table 3:** Specific adverse events during ruxolitinib treatment.

	All patients		IPSS category			
			Low risk		Intermediate-1 risk	
	*n*	%	*n*	%	*n*	%
Total patients	108	100.00	25	100.00	83	100.00
Grade 3 or higher thrombocytopenia^a^	8	7.41	3	12.00	5	6.02
Grade 3 or higher anemia^b^	24	22.22	5	20.00	19	22.89
Ruxolitinib treatment changes due to adverse reactions						
Dose reduction	19	17.59	3	12.00	16	19.28
Temporary therapy interruption	1	0.93	1	4.00	0	0.00
Therapy discontinuation	1	0.93	1	4.00	0	0.00

IPSS, International Prognostic Scoring System.

^a^Defined as a platelet count < 50 × 10^9^/L at any point after ruxolitinib initiation through last ruxolitinib dose.

^b^Defined as hemoglobin < 8 g/dL at any point after ruxolitinib initiation through last ruxolitinib dose.
